# P01-033 – Co-occurance of Crohn’s disease and FMF

**DOI:** 10.1186/1546-0096-11-S1-A37

**Published:** 2013-11-08

**Authors:** A Epstein, I Ben-Zvi, Y Shinar, M Lidar, S Ben-Horin, A Livneh

**Affiliations:** 1 Heller Institute of Medical Research, Departemnt of Medicine F, Sheba medical center, Ramat-Gan, Tel Aviv, Israel; 2 Sackler Faculty of Medicine, Tel Aviv University, Tel Aviv, Isreal; 3 Heller Institute of Medical Research, Isreal; 4 Gastroenterology Unit, Sheba medical center, Ramat-Gan, Israel

## Introduction

There is an increased prevalence of Crohn's disease (CD) in familial Mediterranean fever (FMF). Previous studies found that neither MEFV, nor NOD2/CARD15 may serve as susceptible genes, leading to FMF-CD comorbidity. In addition to NOD2/CARD15 polymorphism, ATG16L1 and IL-23R gene SNPs were also found to predispose to Crohn's disease (CD). The role of these genes in the occurrence of FMF-CD is currently unknown.

## Objectives

To determine the role of polymorphism in NOD2, ATG16L1 and IL-23R genes in FMF-CD, and characterize the clinical correlates of this association.

## Methods

To enrich for CD associated genes with possible effect on the occurrence of FMF-CD, we identified all patients with FMF-CD in our computerized registry of approximately 12,000 FMF patients. All patients were tested for MEFV, NOD2, ATG16L1 and IL-23R relevant gene mutations and completed a questionnaire, detailing the phenotype of their disease. CD diagnosis was established by typical clinical, radiological and endoscopic findings, while a diagnosis of FMF was determined based on our established set of criteria.

## Results

Nineteen patients with FMF-CD were identified. Of them, 17 consented to participate in this study (8 females, 9 males). All patients were of North-African origin. Ten patients (58%) were carriers of the MEFV M694V mutation (5 homozygous). Eight patients (47%) needed biological treatment to control their CD. Two patients (11.7%) had amyloidosis with chronic renal failure. When compared to published patients with CD alone, the FMF-CD group had comparable rate of Gly908Arg NOD2 mutation (19% vs. 9.8%, P=0.2), Thr300Ala ATG16L1 mutation (78% vs. 58%, p=0.14) but significantly increased rate of the rs1004819 polymorphism in IL-23R (61% vs. 38%, p=0.006).

## Conclusion

The rs1004819 IL-23R polymorphism predisposes for the occurrence of FMF-CD. In patients with this comorbidity, the CD appears to be more severe.

## Disclosure of interest

None declared

